# Combining Data from Multiple Sources to Evaluate Spatial Variations in the Economic Costs of PM_2.5_-Related Health Conditions in the Beijing–Tianjin–Hebei Region

**DOI:** 10.3390/ijerph16203994

**Published:** 2019-10-18

**Authors:** Xiya Zhang, Haibo Hu

**Affiliations:** Institute of Urban Meteorology, China Meteorological Administration, Beijing 100089, China; hbhu@ium.cn

**Keywords:** fine particulate matter, health impact, economic cost, spatial assessment

## Abstract

Fine particulate matter, known as PM_2.5_, is closely related to a range of adverse health outcomes and ultimately imposes a high economic cost on the society. While we know that the costs associated with PM_2.5_-related health outcomes are not uniform geographically, a few researchers have considered the geographical variations in these costs because of a lack of high-resolution data for PM_2.5_ and population density. Satellite remote sensing provides highly precise, high-resolution data about how PM_2.5_ and population density vary spatially, which can be used to support detailed health-related assessments. In this study, we used high-resolution PM_2.5_ concentration and population density based on remote sensing data to assess the effects of PM_2.5_ on human health and the related economic costs in the Beijing–Tianjin–Hebei (BTH) region in 2016 using exposure-response functions and the relationship between health and economic costs. The results showed that the PM_2.5_-related economic costs were unevenly distributed and as with the population density, the costs were mainly concentrated in urban areas. In 2016, the economic costs of PM_2.5_-related health endpoints amounted to 4.47% of the total gross domestic product in the BTH region. Of the health endpoints, the cost incurred by premature deaths accounted for more than 80% of the total economic costs associated with PM_2.5_. The results of this study provide new and detailed information that could be used to support the implementation of national and regional policies to reduce air pollution.

## 1. Introduction

Air pollution has significant impacts on health. The World Health Organization (WHO) estimated that, worldwide, three million people died prematurely because of air pollution in 2012 [1]. As a major air pollutant, fine particulate matter with an aerodynamic diameter of ≤2.5 µm, known as PM_2.5_ [2,3], remains suspended in the air for a long time. With a small particle size and large surface area, it is very active and carries various poisonous substances into the body, mainly through the respiratory tract, and penetrates deep into the tracheobronchial and alveolar regions [4,5]. Exposure to PM_2.5_ is associated with various health conditions such as respiratory diseases [6], cardiovascular diseases [7,8], lung cancer [9,10,11], nervous system diseases [12], and congenital heart defects [13]. These negative effects on health inevitably increase the economic costs of health, which then increase the burden on economic development [14]. Therefore, it is important to assess how air pollution impacts on society and economic development and ascertain the health-related economic costs of PM_2.5_.

To date, researchers have estimated the economic cost of health impacts caused by PM_2.5_ in several cities and countries worldwide [15,16,17]. The economic loss caused by PM_2.5_ was estimated to account for 4.7% of the GDP in the Beijing–Tianjin–Hebei (BTH) area of China in 2009 [18]. The health-related economic losses due to PM_2.5_ pollution in Beijing were between USD 0.76 and 1.04 billion in 2014 and between USD 0.68 and 0.99 billion in 2015 [19]. The economic loss attributable to PM_2.5_-related conditions in the Yangtze River Delta of China was USD 3.5 billion in 2010 [20], while, from 2014 to 2016, an average of 1% of the total gross domestic product (GDP) of 190 Chinese cities was given over to air pollution-related health issues [21]. An assessment of the health burden undertaken in Hong Kong indicated that, each year, an average of 2918 premature mortalities were attributable to PM_2.5_ exposure (95% CI: 1288, 4279) from 2001 to 2016 [22]. The preterm births caused by PM_2.5_ produced external costs of USD 4.33 billion in the USA in 2010 [16]. In Skopje, the capital of Macedonia, the social cost of predicted premature mortalities caused by air pollution in 2012 was estimated to be between USD 631.67 and 1629.05 million [23]. 

Most of these studies, by quantitatively assessing the economic costs of specific cities and regions, have shown that the economic costs of PM_2.5_-related health impacts are considerable. Studies have also examined the spatial distribution of economic losses from PM_2.5_-related health impacts. For example, in Beijing, the external costs were higher in the central and southern areas than in the northern districts [24]. The number of deaths attributable to PM_2.5_ in Taiwan was the highest in the southwest, in New Taipei and Kaohsiung [25]. Another study reported that the levels of premature mortality were high in the central and eastern parts of China, and were the highest in Northern China and the Yangtze River Delta region [26]. Yang et al. (2018) assessed the spatial and temporal patterns of economic losses for the period from 2014 to 2016 in 190 Chinese cities [21]. 

Although these studies have estimated the spatial distribution of the economic loss at either the district or national scale, they have assumed that both the exposed population and the PM_2.5_ concentrations were evenly distributed in the studied cities or districts, mainly because of the limited availability of high-resolution PM_2.5_ concentration and population data [21,24,26]. However, if the variations within cities or districts are ignored, any assessment cannot reflect the true situation. Measurements from stationary ambient monitoring sites have been used as surrogates of personal exposure to PM_2.5_, but these sites are limited and are often sparsely and unevenly distributed, which means that it is difficult to collect data continuously over a wide geographical area. Thus, accurate spatial assessments of the economic costs associated with PM_2.5_ pollution require accurate, high-resolution, spatially-resolved predictions of PM_2.5_ exposure and population density. 

Remotely sensed data from satellites may provide a cost-effective method for predicting PM_2.5_ concentrations and for example, satellite-derived aerosol optical depth (AOD) data can be used in areas where there are very few or no monitoring stations [27,28]. In early 2014, the moderate-resolution imaging spectroradiometer (MODIS) team released the Collection 6 AOD products that have a spatial resolution of 3 km, which is considered to be reasonably high [29]. These 3-km AOD products make it possible to reduce PM_2.5_ exposure errors and estimate the spatial distribution of PM_2.5_ concentrations at a high resolution [30,31]. The nighttime lights time-series (NLT) image products of the Defense Meteorological Satellite Program’s Operational Linescan System (DMSP/OLS) also offer good opportunities for detecting the population distribution because they are closely related to human activities [32]. Numerous studies have used NLT products to obtain accurate and detailed information of population distribution at different scales [33,34]. These products can provide high-resolution, accurate information about the spatial distribution of PM_2.5_ concentrations and population density that can be used to assess the economic costs of PM_2.5_-related health impacts. 

Rapid economic development in China has caused severe environmental pollution, including air pollution [35]. While the BTH region is the most important political, economic, and cultural center of northern China, it is also one of the most polluted clusters of cities in China. In 2022, the Winter Olympics will be held in Beijing and Zhangjiakou, Hebei province. With this event approaching, policies need to be developed to effectively control air pollution. There is therefore an urgent need for high-resolution studies related to PM_2.5_, particularly about how the costs of health impacts linked with PM_2.5_ vary spatially. In this study, the spatial distribution of PM_2.5_ concentrations was predicted from MODIS 3-km AOD and the spatial distribution of the population density in the BTH region was modeled from the DMSP/OLS NTL data. Then, the spatial variations in the health-related economic costs of PM_2.5_ in the BTH region were assessed quantitatively by using the spatially highly resolved remote sensing data, the exposure-response function, and the health economic-loss relationship adjusted to the BTH region. The results provide new information that can be used to formulate effective measures to reduce the economic burden associated with air pollution and, at the same time, offer effective technological support for large-scale social activities such as the Winter Olympic Games.

## 2. Materials and Methods

### 2.1. Fine Particulate Matter (PM_2.5_) Data

To date, a variety of empirical models [36,37] and advanced models [28,30,38] have been developed to estimate PM_2.5_ concentrations from satellite-derived AOD products for different parts of the world. In this study, we estimated the PM_2.5_ concentrations of the BTH region in 2016 at a 3-km resolution using multiple remote sensing images and the linear mixed-effects (LME) model [31,39]. The LME model can be used to account for daily variations in the AOD–PM_2.5_ relationship. Daily variations in the relationship between additional meteorological variables and PM_2.5_ were not considered in our model because they led to overfitting of the model [31]. The model structure was expressed as:

(1) $$\begin{array}{cc} {\mathrm{PM}}_{s,t} & =\left(b_{0}+b_{0,t}\right)+\left(b_{1}+b_{1,t}\right)\times {\mathrm{AOD}}_{s,t}+b_{2}\times {\mathrm{PBLH}}_{s,t}+b_{3}\times {\mathrm{RH}}_{s,t}\\ & +b_{4}\times {\mathrm{WS}}_{s,t}+b_{5}\times {\mathrm{PS}}_{s,t}+\varepsilon \;, \end{array}$$

where PM*_s,t_* (μg/m^3^) is the averaged observed PM_2.5_ concentration at a grid cell *s* on day *t*; *b_0_* and *b_0,t_* are the fixed and day-specific random intercepts, respectively; *b_i_* (*i* = 1, 2, ……) represents the fixed slopes for the predictors; *b_1,t_* is the random slope for AOD for each day; AOD*_s,t_* refers to the MODIS AOD value (unitless) at grid cell *s* on day *t*; and PBLH*_s,t_*, RH*_s,t_*, WS*_s,t_*, and PS*_s,t_* are the meteorological parameters at grid cell *s* on day *t*. The meteorological parameters included the planetary boundary layer height (PBLH, m), relative humility (RH, %), wind speed (WS, m/s), and surface pressure (PS, hPa).

**Figure 1:**
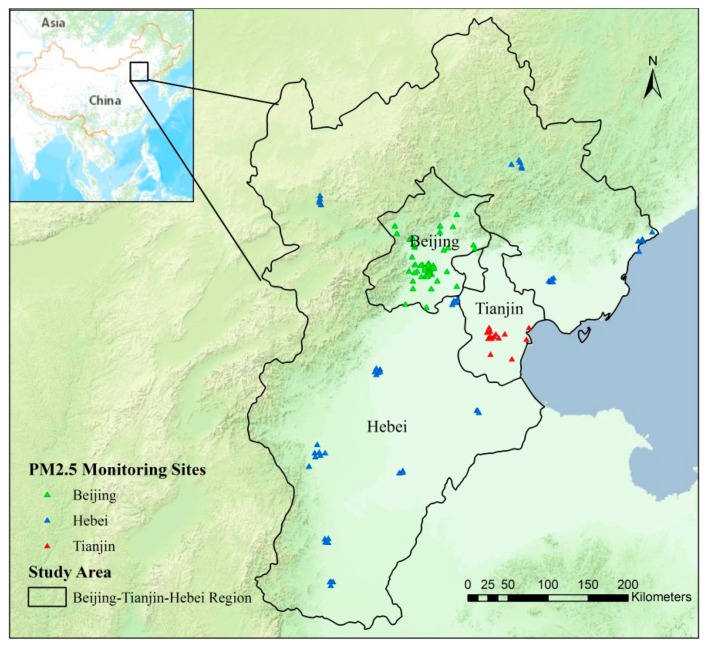
Study area and locations of PM_2.5_ monitoring stations.

The data used in the model included hourly PM_2.5_ concentrations from ground monitoring sites (Figure 1) and the MODIS 3-km AOD and assimilated meteorological data. We obtained PM_2.5_ data for the BTH region for 2016 from China’s Air Quality Online Monitoring and Analysis Platform [40] and the Beijing Municipal Environmental Monitoring Center [41]. We also obtained MODIS Collection 6 Level 2 aerosol products with a spatial resolution of 3 km for 2016 [42]. The data from the 3-km AOD products agreed well with the ground-based data from six Aerosol Robotic Network (AERONET) sites in China [43]. In this study, Aqua (overpass at ~13:30 LocalTime) and Terra (overpass at ~10:30 LocalTime) MODIS AOD data at 550 nm were first combined and averaged to improve the spatial coverage [31]. The 2016 meteorological data were obtained from the Goddard Earth Observing System Data Assimilation Forward Processing (GEOS-5 FP) system. The NASA Global Modeling and ASSimilation Office (GMAO) GEOS-5 FP products, available since April 2012, is the latest version of the GEOS-5 meteorological data, and has a spatial resolution of 0.25° (latitude) × 0.3125° (longitude) in a nested grid that covers China [44]. The hourly PM_2.5_ concentrations and the GEOS-5 FP data from 10:00 to 14:00 local time were averaged as the daytime PM_2.5_ concentrations and meteorological parameters to match the overpass times of the MODIS Terra and Aqua satellites. All data were reprojected using the Albers Equal Area Conic Projection and were resampled to a spatial resolution of 3 km. The data were clipped to match the administrative boundary of the study area. Finally, all the variables for 2016 were matched by grid cell and date for model fitting.

We used the coefficient of determination (R^2^) to indicate the degree of agreement between the predicted and observed PM_2.5_ concentrations. We also applied a 10-fold cross-validation (CV) method to check for potential model overfitting [45] and used R^2^ as an indicator of the agreement.

### 2.2. The Spatial Distribution of the Population Density 

In this study, the spatial distribution of population density was determined with the spatial lag regression model (SLM) based on DMSP/OLS NTL and land cover data. Traditional regression models assume that the observation is mutually independent, which is not valid because of the spatial structure of the data [46]. The SLM method estimates the spatial dependency in regression analysis and provides information about the spatial relationships among the parameters involved in the model [47]. The population density model is expressed as:

(2) $$POP_{i}=\rho \omega POP_{i}+uDN_{i}+\overset{n}{\underset{i=1}{\sum }}\left(a_{k}L_{ik}\right)+\mu ,$$

where *POP_i_* denotes the initial population of the *i*th pixel; *DN_i_* denotes the digital number (DN) of the DMSP/OLS NTL image; *L_ik_* denotes the *k*th land class area of the *i*th pixel; *ρ* is the regression coefficient of the spatial lag variable *ωPOP_i_*; *u* and *a_k_* are the regression coefficients of *DN_i_* and *L_ik_*, respectively; *ω* denotes the spatial weight matrix; and *µ* denotes the vector of error terms that are independent, but not necessarily identically distributed.

Because there were errors associated with the polynomial regression function, there were discrepancies between the initial population estimate and the census data. A proportionality coefficient was built to reduce the errors [48,49,50], as follows:

*k* = Census/*POP*, (3)

where *k* is the proportionality coefficient of the BTH region, Census is all the census data for the BTH region, and *POP* is the modeled initial total population of the BTH region. The modeled initial population of all the pixels was multiplied by the proportionality coefficient, and the spatialized population of each pixel was obtained, as follows:

Est*POP_i_* = k ∗ *POP_i_*, (4)

where Est*POP_i_* is the spatialized population of the *i*th pixel. As in a previous study [51], the mean relative error (MRE) was adopted to evaluate the overall accuracy of the population density estimates as follows:

(5) $$\gamma_{j}=\frac{\left|\bar{POP_{j}}-POP_{j}\right|}{POP_{j}}\times 100\%,$$

(6) $$\mathrm{MRE}=\overset{n}{\underset{j=1}{\sum }}\left|\gamma_{j}\right|/n,$$

where *γ_j_* is relative error of the *j*th county, $\bar{POP_{j}}$ is the estimated population of the *j*th county, *POP_j_* is the census data of the *j*th county, and *n* is the number of counties. 

**Figure 2:**
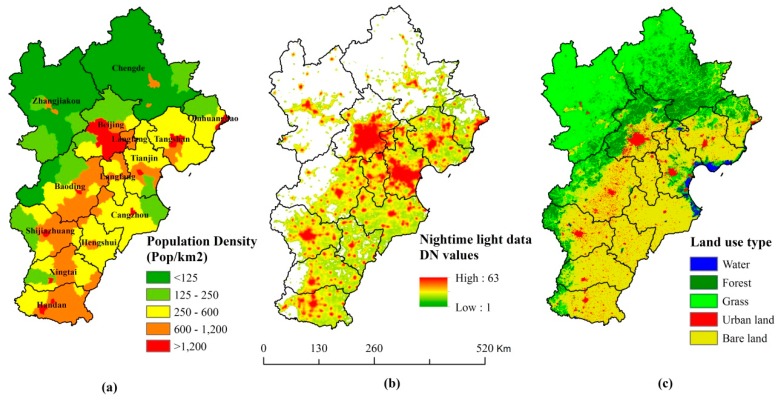
(**a**) Census data, (**b**) DMSP/OLS NTL, and (**c**) land use data of the study area in 2010.

The data sources included three aspects, namely, DMSP/OLS stable NTL data, MODIS land use data, and county-level data from the 6th Census (Figure 2). Because the data from the 6th Census were collected in 2010, we also used NTL data and land use data from 2010. The DMSP/OLS stable NTL data (1-km resolution) values represent the average of all light intensity values observed on cloud-free days during a calendar year from temporally stable sources such as cities, towns, villages, industrial sites, and persistent gas flares [52]; ephemeral events (e.g., fires) were discarded. The DN values of the stable NTL data ranged from 1 to 63 and the background and noise were identified and replaced with values of zero [53]. For land cover, we used the MODIS MCD12Q1 products with a 500-m spatial resolution and the International Geosphere Biosphere Program (IGBP) global vegetation classification scheme. We adopted the IGBP scheme as it is the primary land cover classification scheme used in data from the Land Processes Distribution Active Archive Center of the United States Geological Survey (USGS) [54]. The land cover types of the MCD12Q1 products were amalgamated into five land cover classes, namely, urban land (built-up), forest, grass, water, and bare land (soil) [55,56]. The DMSP/OLS stable NTL data and MCD12Q1 from 2010 were reprojected to the Albers Conical Equal Area projection and were then co-registered and resampled to a pixel size of 1 km. 

Highly accurate population density data of 2010 with a spatial resolution of 1 km were then produced. The population density distribution in 2016 was produced using the annual population growth rate of the cities from 2010 [57,58]. 

### 2.3. PM_2.5_ Health Risk Assessment

We compared various studies published in China and elsewhere in recent years that reported the health impacts caused by exposure to PM_2.5_ [18,24,59,60,61,62,63,64] and established the exposure-response correlation between PM_2.5_ pollution and health effects in the BTH region. We estimated the PM_2.5_ health impacts in this study from mortality data, hospital admission rates for respiratory and cardiovascular diseases, outpatient visits to internal medicine and pediatric practitioners, and co-occurrences of chronic bronchitis, acute bronchitis, and asthma.

Disease and death in the population are probabilistic events that correspond to the Poisson distribution. The linear exposure-response functions (Equations (7) and (8)) were used [65,66]:

(7) $$E_{0i}=\frac{E_{i}}{exp[\beta_{i}*\left(C-C_{0}\right)]},$$

(8) $$HI_{ij}=Pop_{j}\times \left(E_{i}-E_{0i}\right)=Pop_{j}\times E_{i}\times \{1-1/exp[\beta_{i}\times \left(C-C_{0}\right)]\},$$

where *β_i_* is the exposure-response coefficient of health endpoint *i*, *C* is the ambient concentration of PM_2.5_ (μg/m^3^), *C_0_* is the threshold level at which no health effects are yet assumed (μg/m^3^), *E_i_* is the actual morbidity or mortality of the health endpoint *i* under pollution level *C*, *E_*0*i_* is the morbidity or mortality of a certain health endpoint *i* at the threshold concentration level, *HI_ij_* refers to the health impact *i* in city *j* under pollution level *C*, and *Pop_j_* is the exposed population (person) in city *j*. In this study, considering the potential of results in China, we used a baseline concentration (*C*_0_) of 35 μg/m^3^, the threshold suggested by China’s Environmental Protection Ministry [18,24,67,68]. 

The concentration-response coefficients for short-term mortality and morbidity are based on the previously published values listed in Table 1. To maximize the regional accuracy, we prioritized studies carried out in China when developing the exposure-response parameters. The mortality, respiratory hospital admission, and cardiovascular hospital admission parameters mainly refer to the meta-analysis results from Xie et al. (2009) [63] and Aunan and Pan (2004) [61]. The outpatient visits to pediatrics and internal medicine data are from the research about association of air pollution with hospital outpatient visits in Beijing [59]. The data of acute bronchitis, chronic bronchitis, and asthma attack are from the relevant domestic research. Table 2 shows the mortality and morbidity for the health endpoints in the BTH region, making reference mainly to the statistical and research results in the BTH region or across the nation. This information was used to calculate the patient and excess death numbers at health effect endpoints caused by PM_2.5_. 

### 2.4. Quantitative Estimation of the Economic Costs

In this study, the value of a statistical life (VOSL) and the cost of illness (COI) methods were used to estimate the external costs, based on the health impacts. The VOSL is the value of a small change in the risk associated with the death of an unnamed member of a large group [73]. The VOSL represents society’s collective willingness to pay (WTP) [74] for a small reduction in the annual mortality risk of death [75]. In this study, the VOSL method was applied to assess the economic losses in the context of excess deaths and chronic diseases caused by PM_2.5_ [65]. We used data from a contingent valuation (CV) method conducted in Chongqing [65,70,75,76], where the VOSL of a local resident was about USD 34,458 and could increase by USD 14,434 for an annual income increase of USD 144.58. The VOSL of residents in the BTH region in 2016 was calculated as follows:

(9) $${\mathrm{VOSL}}_{j}={\mathrm{VOSL}}_{Cq}\times {\left(\frac{I_{j}}{I_{Cq}}\right)}^{e},$$

where *VOSL_j_* and *VOSL_Cq_* are the VOSL of the city *j* and Chongqing, respectively; *I_j_* and *I_Cq_* are the personal income of the city *j* and Chongqing in 2016, respectively; and *e* is the elastic coefficient of WTP, which was assumed to be 1.0 in this study. The cost of chronic bronchitis was estimated as 0.055 of the VOSL, as suggested by Hammitt and Zhou (2006) [77]. 

The COI method calculates the cost of a disease from the costs of medical treatment and hospitalization, and productivity loss [78]. Based on the health effects calculated above, the associated economic costs were obtained using the following equation:

(10) $${\mathrm{EC}}_{ij}={\mathrm{HI}}_{ij}\times {\mathrm{Cos}t}_{ij},$$

where EC*_ij_* is the economic loss from health impact *i* in city *j* and Cost*_ij_* is its economic cost per case. The values used for the economic costs per case of different health impacts and the literature on which the estimates were based are provided in Table 3. The exchange rate in 2016 was 6.6423 CNY to 1 USD [79].

## 3. Results

### 3.1. Spatial Variations in PM_2.5_ and Population Density

For model fitting, the overall value of R^2^ between the predicted and observed PM_2.5_ concentrations was 0.7495, and the Root Mean Squard Error (RMSE) and Maximum Permissible Error (MPE) were 20.91 μg/m^3^ and 14.55, respectively. The CV of the R^2^ value of the model was 0.7077, which was 0.04 less than that for the model fitting results. The CV of the RMSE value for the model was about 1 μg/m^3^ higher than the model fitting results, which suggests that the model was not substantially overfitted.

**Figure 3:**
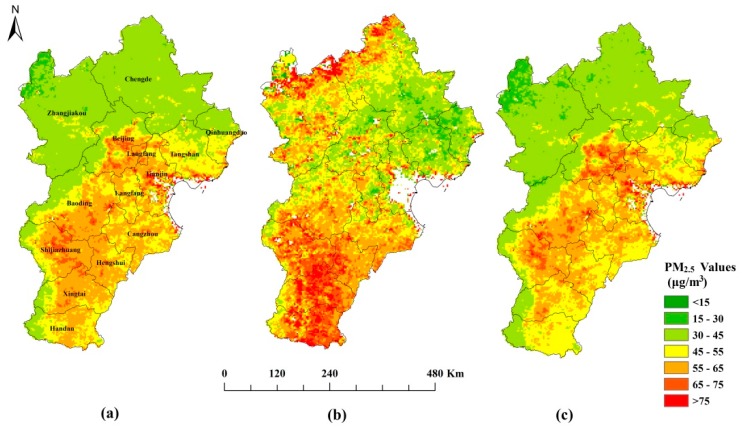
Spatial distributions of the (**a**) annual mean PM_2.5_ concentration, (**b**) cold season mean PM_2.5_ concentration, and (**c**) warm season mean PM_2.5_ concentration.

The spatial distribution of PM_2.5_ and the mean distributions of PM_2.5_ for 2016 for two seasons across the BTH region are shown in Figure 3. Because there were limited satellite data for winter (January, February, and December), the data were grouped into two seasons, namely, warm for the period from 15 April to 14 October and cold for the period from 15 October to 14 April. The blank areas in Tianjin in Figure 3 reflect that there is no predicted PM_2.5_ due to the lack of satellite AOD data. Spatially, there was a southeast-to-northwest gradient in the predicted concentrations of PM_2.5_. The concentrations were high in Beijing and in the south of Hebei province, and were low in the north of Hebei province. There were distinct seasonal variations in PM_2.5_ in the BTH region, and, apart from Beijing, the concentrations were the highest in the cold season and the lowest in the warm season. The abnormal seasonal variation in Beijing areas is due to the lack of satellite AOD. Both Terra and Aqua AOD data are not available in some days in cold season, especially in the urban center areas of Beijing. The predicted PM_2.5_ concentrations in the warm season and the cold season ranged from 8 to 190 μg/m^3^, and from 2 to 300 μg/m^3^, respectively. This seasonal variation reflects both the meteorological conditions and local emission sources.

**Figure 4:**
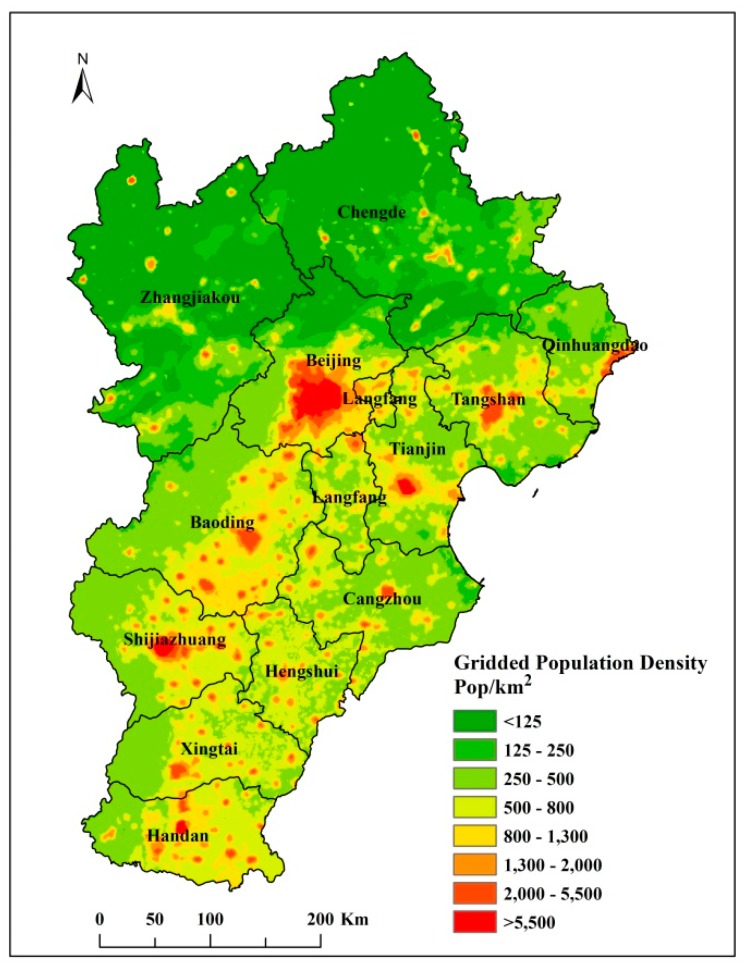
The population density across the BTH region in 2016.

The MRE calculated for the validation population result was about 34.62%, which proved that the estimates of the population density were relatively high. The simulated population density is presented in Figure 4. The BTH region is one of the most densely populated regions in China, with population densities exceeding 500 people/km^2^. The population densities were very obviously higher in the urban areas (generally exceeded 1000 people/km^2^) than in the rural areas (generally less than 250 people/km^2^).

### 3.2. Health Effects Linked to PM_2.5_

The health impacts calculated from a baseline concentration of 35 μg/m^3^ are shown in Table 4. Our results suggest that a PM_2.5_ pollution event of this magnitude would result in 89,005 acute deaths from all causes in the BTH region, with 15,934 deaths in Beijing, 11,004 deaths in Tianjin, and 62,067 deaths in Hebei. Of the health endpoints, the number of cases of outpatient visits to internal medicine was the highest in Beijing and Tianjin, followed by acute bronchitis and outpatient visits to pediatrics. For Hebei, the number of cases of acute bronchitis was the highest of all health endpoints, followed by outpatient visits to internal medicine and pediatrics. These effects probably reflect both the PM_2.5_ pollution and population distribution. While the mean PM_2.5_ concentrations of the three regions were similar (about 70 µg/m^3^), the number of cases for each health impact was much greater in Hebei than in Beijing and Tianjin, because the total population of Hebei (about 7.47 million) was much larger than the population of either Beijing (about 2.173 million) or Tianjin (about 1.562 million). Generally, as the PM_2.5_ pollution and population increased, the health effects in the BTH region also increased.

**Figure 5:**
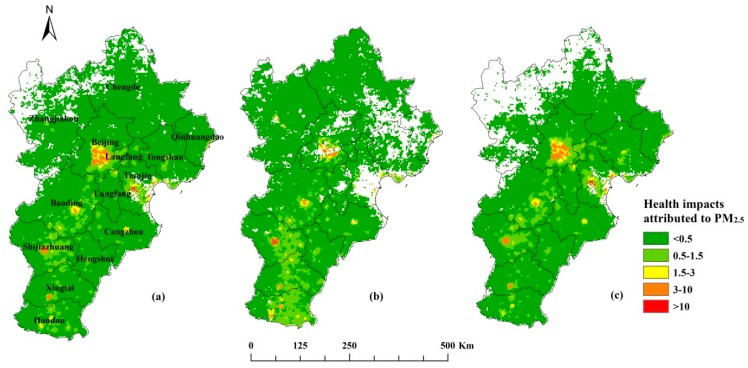
Spatial distribution of the health impacts associated with PM_2.5_ in (**a**) 2016, and during the (**b**) cold season and (**c**) warm season of 2016 (unit cases).

The spatial distribution of the calculated health impacts of PM_2.5_ in the BTH region are shown in Figure 5. The blank areas of the figure represent the areas where the PM_2.5_ concentration is lower than 35 μg/m^3^ and there are no health impacts. From the figure, we can see there was considerable spatial heterogeneity, and that more residents were affected by the PM_2.5_ pollution in central Beijing, Tianjin, and the southern districts of Hebei. The health impacts were also directly proportional to the population size. In the cold season, PM_2.5_ pollution was high to the northwest of Zhangjiakou and Chengde in the Hebei province (Figure 3), but the health impacts in these districts were smaller than those in central Beijing because of the differences in population distribution across these districts (Figure 4). The spatial patterns of health impacts in different seasons, based on the same population density (shown in Figure 5a–c), were similar to the patterns in the PM_2.5_ concentrations (Figure 3).

### 3.3. Economic Costs of PM_2.5_

The economic costs calculated for all health endpoints and all-cause mortality for a baseline PM_2.5_ concentration of 35 μg/m^3^ are shown in Table 5. Of the three areas, the external costs of PM_2.5_ were the highest in Hebei and lowest in Tianjin, where it was around 23% of the cost in Hebei. Economic losses due to all-cause mortality accounted for about 81% of the total external costs of PM_2.5_ pollution. Chronic bronchitis accounted for most of the cost of morbidity health impacts. 

The ratio of the economic cost to GDP (Ecal/GDP) describes the economic burden of the health impacts linked to PM_2.5_ pollution and thus, it is an important parameter for formulating effective policies to maintain sustainable economic development [76]. The current economic development means that the values of Ecal/GDP are relatively high for this study. The economic cost of the health effects related to PM_2.5_ pollution in Beijing was 15.16 billion USD, around 3.9% of the GDP, while the economic costs of the health effects of PM_2.5_ pollution were 2.5% and 6% of the GDP in Tianjin and Hebei, respectively. These results suggest that the economic cost of health impacts related to PM_2.5_ has increased the economic burden in the BTH region.

**Figure 6:**
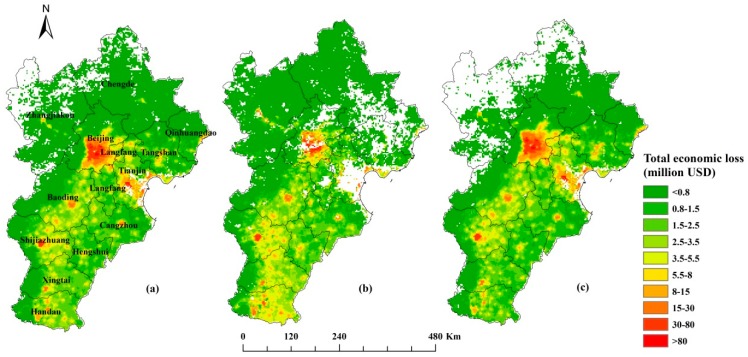
Spatial distribution of the total economic costs associated with PM_2.5_ in (**a**) 2016, and in (**b**) the cold season and (**c**) the warm season in 2016.

The spatial distribution of the total economic cost associated with PM_2.5_ in 2016 and changes in the spatial distribution over the seasons are presented in Figure 6. The blank areas of the figure represent the areas where there was no economic cost linked to PM_2.5_. The blank areas in Tianjin in Figure 6b reflect the lack of PM_2.5_ data for cold season, which we have not discussed. The economic losses reflected the population density (Figure 4) and were concentrated in the urban areas and limited in the suburbs. The urban areas of Beijing, Tianjin, Shijiazhuang, Baoding, Xingtai, and Handan had high health-related economic losses because of PM_2.5_. The ranking was similar in different seasons and highly impacted areas were concentrated in the south of the BTH region, while the lower-ranked areas were mainly concentrated in the northern part of the BTH region. In the north of Hebei province, there was no economic cost in the warm season and the economic costs were low in the cold season (blank areas in Figure 6). In Chengde and Qinhuangdao in the southeast, the economic costs because of PM_2.5_ were lower in the cold season than in the warm season. The seasonal variation in the spatial distribution of the economic costs associated with PM_2.5_ mainly reflects the seasonal variations in PM_2.5_. In general, PM_2.5_ pollution and population distribution together account for the variable economic costs, and as the PM_2.5_ and population density increased, the economic costs also increased. 

## 4. Discussion

We assessed the spatial variation in the economic costs of PM_2.5_-related health impacts in 2016 in the BTH region. Overall, the annual economic cost of these illnesses in the BTH region is high, especially in the central and southern areas of Beijing, and in the urban areas of Tianjin, Shijiazhuang, and Baoding. The high costs in these areas reflect pollution emissions, meteorological conditions, and population density. The areas with the most difference between the cold season and warm season were concentrated in Zhangjiakou, Chengde, and the southern parts of Hebei. The variability in the spatial distribution between the seasons reflects the seasonal variations in the PM_2.5_ concentrations. 

The results of this study show that more than 2.3 million people suffered from health impacts related to PM_2.5_. These health impacts resulted in more than 15,000 premature deaths, representing about 0.07% of the 21.73 million residents in Beijing in 2016. In Tianjin, 1.6 million people suffered health impacts and 11,000 or 0.07% of the 15.62 million residents died prematurely. In Hebei, 7.8 million people suffered health impacts and 62,000 or 0.08% of the 74.70 million residents died prematurely. The most common health endpoints were outpatient visits to internal medicine and acute bronchitis. These results are generally consistent with those of previous studies; for example, Xie et al. (2011) reported that about 0.08% of the total population in the Pearl River Delta region in China died prematurely from PM_2.5_-related illnesses [80], while Apte et al. (2015) from their assessment of the health impacts of PM_2.5_ on people of all ages in China found that premature deaths accounted for 0.09% of the total population in 2013 [81].

We calculated that the total external costs of PM_2.5_-related health impacts in Beijing, Tianjin, and Hebei were 15.16, 6.8, and 29 billion USD, which was equivalent to around 3.9%, 2.5%, and 6% of the GDP of these regions in 2016, respectively. These findings are similar to those from other studies. For example, Lv and Li (2016) reported that the economic losses from health impacts as a result of PM_2.5_ pollution accounted for 2.16% (VSL) of the regional GDP in the BTH region in 2013 [67], while other researchers reported that the economic costs of PM_2.5_ pollution accounted for 4.98% and 3.79% of Beijing’s GDP in 2008 and 2012, respectively [66]. These estimates of the economic losses may vary depending on the PM_2.5_ concentration levels, baseline levels, health impact assessment models, exposure-response coefficients, and the choice of health endpoints. 

We used high-resolution population data and satellite-derived PM_2.5_ data to explore the spatial distribution of economic costs from PM_2.5_-related health impacts. Because of the limited availability of high-resolution PM_2.5_ and population data, previous studies only estimated the economic costs at the district scale. However, the population density and PM_2.5_ concentrations vary considerably within cities and districts. Our study, therefore, is significant because we estimated the district-level and within-district economic costs related to air pollution in the BTH region. Our results build on those obtained from previous studies and provide more detailed information for policymakers about areas that should be given priority when developing strategies to control and prevent air pollution in this era of increased environmental protection in China. 

However, there are some limitations in this analysis. First, it is difficult to consider all the possible health endpoints related to PM_2.5_ and obtain the associated data and information [24]. Furthermore, the biological evidence for some health impacts is still not certain [82]. Hence, we did not include some important potential PM_2.5_ health impacts such as diabetes and mental and behavioral disorders, which could impact on a person’s ability to work or engage in routine daily activities, and could represent significant additional health and economic burdens on social services. Second, in our study, Aqua and Terra MODIS AOD data were combined to improve spatial coverage, but neither Terra nor Aqua AOD data are available for some days because of cloud contamination, especially in the cold season. The predicted PM_2.5_ concentrations are affected by the missing values of the satellite AOD data, which require more accurate PM_2.5_ spatial distributions in the study area. Third, the population density data also has its limitations. Although the estimates of the population density in 2010 were relatively high, the usage of annual population growth rate of the cities from 2010 to 2016 to produce the population density distribution in 2016 may introduce some errors, which may affect the results. Fourth, we used a baseline PM_2.5_ concentration of 35 μg/m^3^, as suggested by the Chinese Environmental Protection Ministry, and considered a sensitive parameter for both health impacts and estimating external costs [24]. In future studies, the WHO-recommended baseline of 10 μg/m^3^ could also be used to estimate the external costs and the results calculated from both the baseline concentrations could be compared. 

Despite these limitations, the results from this study can still be used to show the levels of air pollution and the district-level and within-district spatial variations of economic losses from PM_2.5_-related health impacts in the BTH region. However, to curb the health-related economic costs related to PM_2.5_, the PM_2.5_ concentrations need to be reduced further. 

## 5. Conclusions

We combined simulated high-resolution population density data and satellite-derived PM_2.5_ concentration data and developed maps of the spatial distributions of economic losses related to multiple health impacts caused by PM_2.5_ pollution in 2016 in the BTH region. The results showed that the PM_2.5_-related economic losses of health impacts in the BTH region were enormous, and were close to 4.47% of the BTH’s regional GDP in 2016. We also found that the economic costs were unevenly distributed and as observed for population density, the costs were concentrated in urban areas and low in the suburbs. The losses were the greatest in Beijing, Tianjin, and cities in the southern Hebei province such as Shijiazhuang, Baoding, Xingtai, and Handan, and reflect the spatial distribution of the PM_2.5_ pollution. Priority should be given to controlling the PM_2.5_ pollution in these central and southern areas. There are some limitations associated with the economic losses estimated in this study because of data and research limitations. For instance, we did not include all PM_2.5_-related health impacts in the assessment and we used a static population exposure model in the study. These limitations should be addressed in future studies. Regardless of these limitations, the results from this study can still be used to enhance our fundamental understanding of the effects of PM_2.5_ pollution on our health and economy. This study highlights important information that can be used to reduce the health risks and economic burdens associated with PM_2.5_. It should also be useful for policymakers and should support the design of new policies to prevent and control air pollution.

## Figures and Tables

**Table 1:** PM_2.5_ exposure-response coefficients for the chosen health endpoints.

Health Impact	Exposure-Response Coefficients 95% CI	Reference
All-cause mortality	0.0040 (0.0019, 0.0062)	[63]
Respiratory hospital admission	0.0109 (0, 0.0221)	[60,61,63] [60,61,63]
Cardiovascular hospital admission	0.0068 (0.0043, 0.0093)	[60,61,63]
Outpatient visits to pediatrics	0.0056 (0.0020, 0.09)	[59]
Outpatient visits to internal medicine	0.0049 (0.0027, 0.07)	[59]
Acute bronchitis	0.0790 (0.027, 0.13)	[69]
Chronic bronchitis	0.01009 (0.00366, 0.01559)	[18]
Asthma attack	0.02100 (0.0145, 0.03)	[70]

**Table 2:** Mortality and morbidity at the health endpoints used in the analysis (95% CI).

Health Impact	E_Beijing_	E_Tianjin_	E_Hebei_	Reference
All-cause mortality	5.20‰	5.54‰	6.36‰	[58]
Respiratory hospital admission	2.03%	1.47%	2.11%	[71]
Cardiovascular hospital admission	1.57%	1.14%	1.64%	[71]
Outpatient visits to pediatrics	77.00‰	52.58‰	96.10‰	[71]
Outpatient visits to internal medicine	22.20%	31.70%	21.80%	[71]
Acute bronchitis	3.80%	3.80%	3.80%	[18,72]
Chronic bronchitis	0.69%	3.80%	3.80%	[18,72]
Asthma attack	0.94%	0.94%	0.94%	[18,72]

**Table 3:** Economic costs per case of different health effects (USD).

Health Impact	EC_Beijing_	EC_Tianjin_	EC_Hebei_	Method	References
All-cause mortality	775,333.85	502,461.50	383,481.63	VOSL	[58]
Respiratory hospital admission	1096.17	1096.17	1096.17	COI	[71]
Cardiovascular hospital admission	2595.54	2595.54	2595.54	COI	[71]
Outpatient visits to pediatrics	69.28	45.06	32.35	COI	[71]
Outpatient visits to internal medicine	69.28	45.06	32.35	COI	[71]
Acute bronchitis	376.39	304.86	211.21	COI	[18,67]
Chronic bronchitis	42,643.36	27,635.38	21,091.49	VOSL	[77]
Asthma attack	277.06	224.41	155.39	COI	[67]

**Table 4:** Estimates of the cases (and 95% CI) attributable to PM_2.5_ pollution (C_0_ = 35 μg/m^3^) (Unit: Thousand cases).

Health Impact	Beijing	Tianjin	Hebei
All-cause mortality	15.934 (7.871, 23.718)	11.004 (5.413, 16.447)	62.067 (30.566, 92.676)
Respiratory hospital admission	149.415 (0, 250.340)	70.832 (0, 120.835)	500.378 (0, 849.737)
Cardiovascular hospital admission	77.810 (51.511, 101.725)	36.617 (24.128, 48.089)	259.152 (170.962, 339.954)
Outpatient visits to pediatrics	320.725 (122.453, 484.648)	142.390 (53.991, 216.506)	1277.715 (485.322, 1939.757)
Outpatient visits to internal medicine	819.571 (470.404, 1126.711)	759.872 (434.312, 1048.723)	2,566.449 (1468.419, 3538.570)
Acute bronchitis	784.711 (529.764, 819.832)	553.107 (356.541, 586.417)	2659.836 (1735.294, 2808.604)
Chronic bronchitis	48.028 (19.580, 67.412)	31.481 (12.684, 44.600)	154.244 (62.332, 218.013)
Asthma attack	112.297 (86.531, 138.935)	74.929 (57.147, 93.883)	365.481 (279.469, 456.461)

**Table 5:** Average total economic cost (billion USD) as a proportion of the BTH’s average GDP.

Health Impact	Economic Cost (95%CI)		
	Beijing	Tianjin	Heibei
All-cause mortality	12.35 (6.10, 18.39)	5.53 (2.72, 8.26)	23.80 (11.72, 35.54)
Respiratory hospital admission	0.16 (0, 0.27)	0.08 (0, 0.13)	0.55 (0, 0.93)
Cardiovascular hospital admission	0.20 (0.13, 0.26)	0.10 (0.06, 0.12)	0.67 (0.44, 0.88)
Outpatient visits to pediatrics	0.02 (0.01, 0.03)	0.01 (0.002, 0.01)	0.04 (0.02, 0.06)
Outpatient visits to internal medicine	0.06 (0.03, 0.08)	0.03 (0.02, 0.05)	0.08 (0.05, 0.11)
Acute bronchitis	0.30 (0.20, 0.31)	0.17 (0.11, 0.18)	0.56 (0.37, 0.59)
Chronic bronchitis	2.04 (0.83, 2.87)	0.86 (0.35, 1.23)	3.25 (1.31, 4.60)
Asthma attack	0.03 (0.02, 0.04)	0.02 (0.01, 0.02)	0.05 (0.04, 0.07)
Total loss	15.16 (7.34, 22.26)	6.80 (3.28, 10.01)	29.00 (13.95, 42.79)
GDP	386.45	269.26	482.81
Total loss/GDP	3.90%	2.52%	6.00%
